# Occurrence and Diversity of Cyanotoxins and Retinoid Compounds in Antarctic Microbial Mats: Evidence From James Ross Island

**DOI:** 10.1111/1758-2229.70321

**Published:** 2026-03-31

**Authors:** Luděk Sehnal, Lucie Bláhová, Marie Smutná, Ondřej Mikeš, Pavel Babica, Klára Hilscherová

**Affiliations:** ^1^ RECETOX, Faculty of Science, Masaryk University Brno Czech Republic; ^2^ Department of Experimental Biology Faculty of Science, Czech Collection of Microorganisms, Masaryk University Brno Czech Republic; ^3^ Department of Experimental Phycology and Ecotoxicology Institute of Botany of the Czech Academy of Sciences Brno Czech Republic

**Keywords:** Antarctic, cyanotoxins, EDCs, microorganisms, retinoids, saxitoxin

## Abstract

Antarctic ecosystems, though extreme, harbour diverse microbial communities dominated by cyanobacteria, which serve as crucial primary producers. Whilst the occurrence of cyanotoxins has been extensively studied in temperate climates, limited research has focused on their presence in polar regions. In this study, we investigated the distribution and diversity of cyanotoxins across various aquatic ecosystems on James Ross Island, Antarctica. Using targeted LC–MS/MS analysis, we detected microcystins, nodularin, cylindrospermopsin, and, for the first time in the Antarctic region, the neurotoxin saxitoxin, albeit at concentrations below quantifiable levels. Our findings also reveal the widespread presence of retinoids in microbial mats, with potential endocrine‐disruptive properties, marking the first report of these metabolites in Antarctic ecosystems. This study highlights the ecological importance of cyanobacterial metabolites in Antarctic environments and raises concerns about their potential effects on local wildlife and water quality, particularly in the context of climate change. The findings underscore the need for continued monitoring and molecular studies to elucidate the sources and ecological roles of cyanotoxins in polar regions.

## Graphical abstract

Occurrence and distribution of cyanotoxins and retinoids across different Antarctic ecosystems at James Ross Island. These ecosystems harbour diverse microbial communities dominated by autotrophic cyanobacteria and algae, which can produce hazardous compounds for animals.

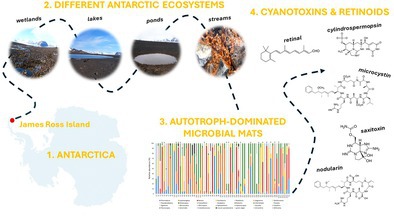

## 1 Introduction

Most of the life in the terrestrial Antarctic is limited to aquatic refugia such as shallow lakes and ponds, wetlands, streams, wet rocks, or cryconites. Only a narrow spectrum of organisms can survive or even thrive in these ecosystems under such harsh environmental conditions. One dominant and well‐adapted group of organisms with very crucial functions in Antarctic ecosystems is cyanobacteria. These phototrophic prokaryotic organisms play an important role as primary producers and are key components of microbial mats across various aquatic ecosystems in Antarctica (Vincent and Quesada 2012; Velichko et al. 2021; Vincent 2000; Jungblut et al. 2010; Sehnal 2015). Importantly, cyanobacteria have been described as prolific producers of various specialised metabolites that they use to communicate with their surrounding environment (de Freitas et al. 2024; Leão et al. 2012). Particularly, those cyanobacterial metabolites that exhibit toxic properties gained the biggest attention and were named after their producers, cyanotoxins.

Historically, the research on cyanotoxins has been largely concentrated on more temperate climates, where blooms of toxin‐producing cyanobacteria have been extensively documented (Blahova et al. 2021). The most problematic and studied cyanotoxins include hepatotoxic microcystins, nodularins, cylindrospermopsins, and two groups of neurotoxic cyanobacterial metabolites—anatoxins and saxitoxins (Svirčev et al. 2019). These toxins have been identified as potent biohazards with the potential to contaminate drinking water sources, recreational water bodies, and other aquatic ecosystems; thus, adversely affecting not only humans but also animals within these ecosystems (Igwaran et al. 2024; Codd et al. 1999; Ndungu et al. 2025; Kubickova et al. 2019). In the past two decades, researchers have also investigated cyanobacterial production of these toxins in both Arctic and Antarctic ecosystems (Chrapusta et al. 2015; Wood et al. 2008; Kleinteich et al. 2014; Jungblut et al. 2006). A set of studies measuring the concentration of different cyanotoxins documented the presence of different variants of microcystin, nodularin, and cylindrospermopsin in microbial mats dominated by cyanobacteria from both Arctic and Antarctic freshwater ecosystems, whilst neurotoxic anatoxin and saxitoxin were detected only in Arctic ecosystems (de Freitas et al. 2024; Cirés et al. 2017). Moreover, besides these groups of cyanotoxins, a new group of possible toxic compounds from cyanobacteria emerged with the identification of endocrine‐disrupting effects of several classes of cyanobacterial metabolites. In general, these compounds are called endocrine‐disrupting chemicals (EDCs), and cyanobacteria have been shown to produce retinoids and/or oestrogens that can interact with hormone signalling pathways in animals (Jonas et al. 2015; Pípal et al. 2022; Smutná et al. 2023). Nevertheless, there is no available information about EDCs from microbial communities in polar regions.

Although Antarctica is an extreme environment with conditions that limit permanent human presence, it supports diverse microbial and animal life. There are vertebrate animals such as seals or birds, and numerous invertebrates thriving in the extreme conditions of Antarctica, which can be threatened by cyanobacterial metabolites. As climate change continues to exert its influence on polar ecosystems (Post et al. 2019), including Antarctica isolated by the Antarctic convergence, understanding the dynamics of cyanotoxins becomes imperative for predicting and mitigating potential ecological consequences. Besides cyanobacteria and other prokaryotes, it has been shown that Antarctic microbial communities across different aquatic ecosystems are comprised of diverse microbial eukaryotes (Jungblut et al. 2012). Those eukaryotic organisms are in close interkingdom interaction with prokaryotes and cyanobacterial metabolites, including cyanotoxins, which can play a significant role in these relationships (Omidi et al. 2021).

The current knowledge about cyanotoxins in Antarctica is limited to lake ecosystems, and only nodularin, microcystin, and cylindrospermopsin were previously detected, whilst no information about any EDCs, including retinoids, is available from Antarctic microbial communities. Through an analysis of different types of microbial mats dominated by cyanobacteria, algae, and diatoms, this research aims to investigate the distribution, diversity, and level of cyanotoxins and retinoids across different types of aquatic ecosystems at James Ross Island, Antarctica. Information about the distribution and diversity of broader spectra of cyanotoxins and cyanobacterial metabolites can bring important data regarding the assessment of the potential risk of these metabolites in light of climate change, which currently affects the whole planet, including Antarctica.

## 2 Experimental Procedures

### 2.1 Sample Collection, Storage and Retrieval

Fieldwork in Antarctica was undertaken on the Ulu Peninsula, which represents the northern part of James Ross Island (JRI), where the Czech Antarctic station, Johann Gregor Mendel Station, is located (S 63°48′02″ W 57°52′57″). Mean daily air temperatures above 0°C occur only from December to February (Hrbáček et al. 2016). During an expedition to JRI (January–February 2015), 67 samples of microbial mats were collected from different types of environments, including 11 lakes, 12 ponds, 29 streams, 1 wet rock, 5 wetlands, and 9 seepages (Figure 3).

Environmental conditions at sampling times, along with GPS coordinates, were recorded (Table S1). The samples were transferred to a sterile flask and stored at −20°C at JRI on the day of collection. For morphological determination of cyanobacteria in samples, aliquots from each sample were prepared and fixed in 5% formaldehyde and stored at −20°C. The samples were transported to the Czech Republic in a thermo‐cooling box with cooling pads (over a 48‐h period) and then frozen (−20°C) until processing.

### 2.2 Sample Processing

Initially, each sample was divided into two parts. The first part of the sample (1/5 of the total sample amount), intended for light microscopy inspection, enrichment, and isolation of cyanobacterial strains, was stored at −20°C. Storage at freezing temperatures before enrichment and isolation is possible only for cyanobacteria from polar regions, which have developed several cold‐adaptive strategies (osmoprotectants and maintenance of membrane fluidity) to endure both long‐term and short‐term freezing. The second part of the sample (4/5 of the total sample amount), intended for the extraction of DNA and organic compounds, was freeze‐dried for 2 days and ground into powder in liquid nitrogen using a mortar and pestle. The powdered sample was stored at −70°C before subsequent extractions.

### 2.3 Chemical Extraction

**FIGURE 1:**
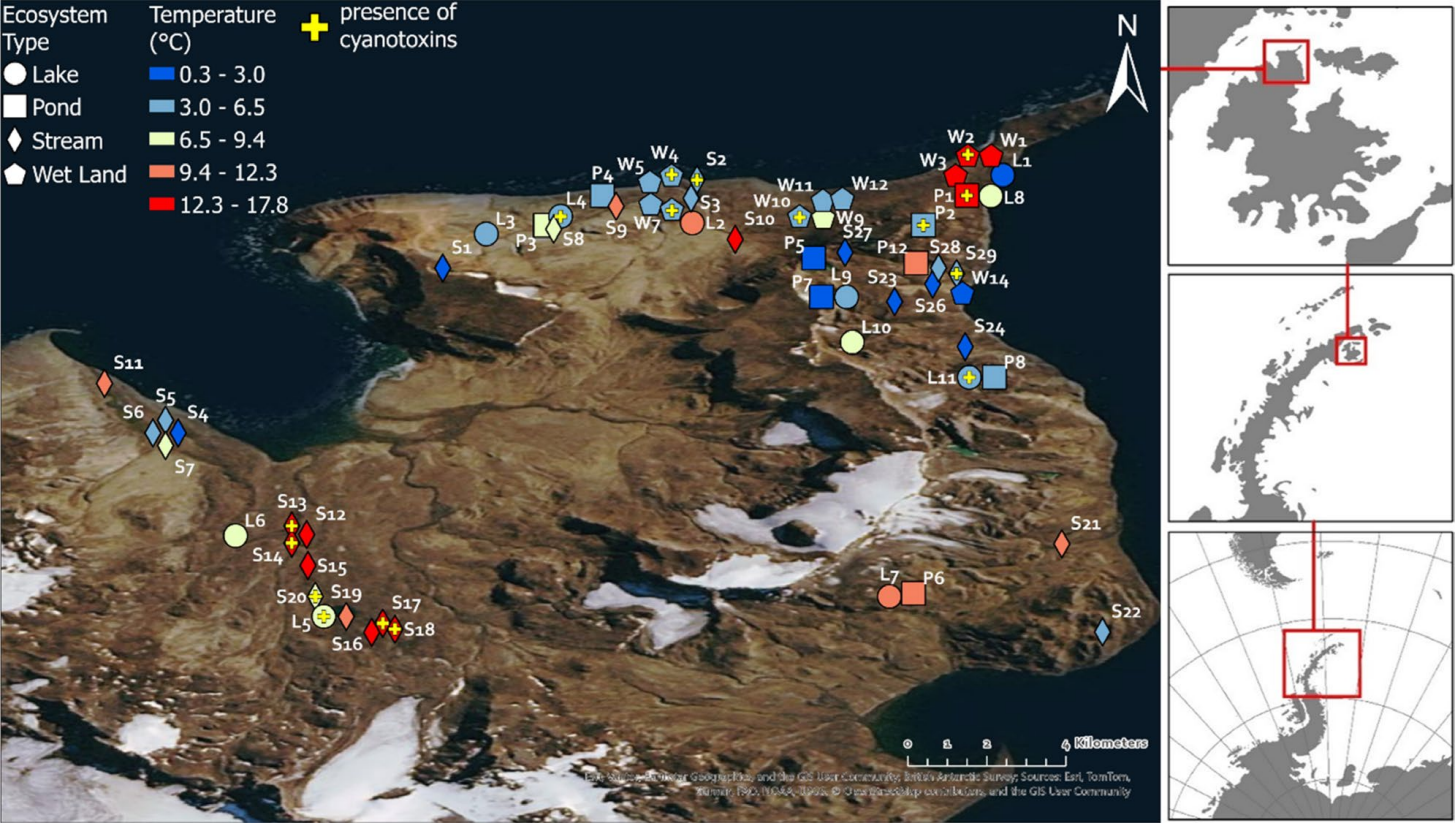
Map of James Ross Island and types of sampling sites with indicated presence of cyanotoxin and localities coloured based on temperature gradient (maps coloured based on conductivity and pH are shown in Figures S1 and S2, respectively). Selected site markers have been offset from their exact positions to avoid overlap on the map. Accurate coordinates for each sampling site are provided in the accompanying sample table (Table S1).

All samples were extracted using the protocol published in a previous study (Javůrek et al. 2015) (Figure 1).

### 2.4 Taxonomic Classification

The samples were taxonomically classified using light microscopy. An overview of the morphological traits of cyanobacteria (Komárek and Anagnostidis 2007) was used for the taxonomic classification of collected samples. For taxonomic analysis, three 2.5 mm × 2.5 mm pieces were cut out from each microbial mat and homogenised in 1 mL of water. Cell counting was carried out using an Olympus BX51 light microscope (Olympus, Japan) and a Bürker chamber. For each sample, at least 300 individuals were identified to the taxonomic level of genera, and every cell in the filament was counted as an individual. The relative abundance of each taxon was expressed as the percentage of the total count (Loza et al. 2013; Zupančič et al. 2021).

### 2.5 LC–MS/MS Analysis

A detailed description of analytical methods for the detection of both retinoids and cyanotoxins is given in the Supporting Informations (S2, S3), together with MS parameters for analysed retinoids and cyanotoxins (Tables S4 and S5). The method for retinoid detection was used for analysis of all‐*trans*‐retinoic acid (ATRA), retinal, 9‐*cis*‐retinoic acid (9‐cis‐RA), 13‐*cis*‐retinoic acid (13‐cis‐RA), 9‐*cis*‐keto‐retinoic acid (9‐cis‐keto‐RA), 13‐*cis*‐keto‐retinoic acid (13‐cis‐keto‐RA), 4‐keto‐all‐*trans* retinoic acid (4‐keto‐ATRA), 4‐keto‐retinal, all‐*trans*‐5,6‐epoxy‐retinoic acid (5,6‐epoxy‐ATRA) and 4‐hydroxy‐all‐*trans*‐retinoic acid (4‐OH‐ATRA). The method for cyanotoxin detection was used for analysis of microcystins (‐LA, ‐LF, ‐LR, ‐LW, ‐LY, ‐RR, ‐YR, ‐WR), anatoxin (ATX), homo‐anatoxin (HATX), cylindrospermopsin (CYN), deoxy‐cylindrospermopsin (deoxy‐CYN), nodularin (NOD), and saxitoxin (SXT). Quality assurance and quality control samples, including blanks, model biomass extracts (in‐house reference material with known level of cyanotoxins and retinoids) were repeatedly analysed. Quality control samples were analysed after every 20 biomass extracts and repeatability was acceptable (RSD < 15%). Three procedural blanks were analysed in each analytical run with concentrations below LOD.

## 3 Results

### 3.1 Concentrations and Diversity of Cyanotoxins and Retinoids Across Different Microbial Communities

Cyanotoxins were detected in 18 different samples, with various forms of microcystin detected in samples from 16 localities, nodularin at 2 localities, saxitoxin at 2 localities and 7‐deoxy‐cylindrospermopsin at 1 sample (Table 1). The concentration of microcystins varied from concentrations below the limit of detection (LOD = 1.25 ng/g dm) to 37.1 ng/g dm (microcystin‐LR at L5). The most abundant and frequent microcystin was microcystin‐LR, which was detected at 11 localities. However, microcystin‐LR was not necessarily the predominant structural variant detected. For example, W8, which exhibited the second‐highest total concentration of microcystins at 34.6 ng/g dry mass (dm), was dominated by microcystin‐WR at 21.6 ng/g dm, along with the presence of microcystin‐YR. Nodularin was detected at a concentration of 4.85 ng/g dm at W4, whilst its concentration at S18 was below the limit of quantification (LOQ). Saxitoxin and deoxy‐cylindrospermopsin were detected only at concentrations between LOD and LOQ. Complete data on cyanotoxin concentrations are shown in Table S2.

**TABLE 1:** The concentration of cyanotoxins (ng/g dm) in Antarctic microbial mats.

	MC‐LR	MC‐LW	MC‐LY	MC‐RR	MC‐YR	MC‐WR	Nodularin	Saxitoxin	deoxyCYN
L4	1.7								
L5	37.1								
L11				1.4					
P1	2.0								
P2	2.5				*				
S2	4.8	7.5	6.7						
S13		*							
S14	3.0							*	
S17		4.1						*	
S18	1.3						*		
S20	2.7								
S25	1.3								
S29				3.4					
W2	3.8								
W4							4.9		
W6									*
W8					13.0	21.6			
W10	1.3								
*LOD*	*0.5*	*1.25*	*0.5*	*0.5*	*1.25*	*1.25*	*0.5*	*1.25*	*1.25*
*LOQ*	*1.25*	*2.5*	*1.25*	*1.25*	*2.5*	*2.5*	*1.25*	*2.5*	*2.5*

*Note:* Asterisk (*) indicates that the compound was detected but not quantified (falling between LOD and LOQ). Only samples with detected cyanotoxins are shown.

Abbreviations: L, lake; P, pond; S, stream; W, wetland.

**FIGURE 2:**
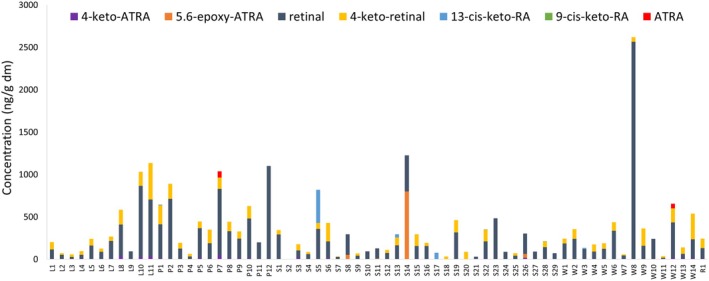
The concentrations of retinoids in samples from Antarctic microbial mats. The concentrations of individual retinoids are expressed in ng/g dm. All analysed retinoids had the same LOD (1.25 ng/g dm) and LOQ (2.5 ng/g dm). For details on sampling sites, see Figure 1 and Table S1. L—lake, P—pond, S—stream, W—wetland, R—wetrock.

A variety of retinoids were detected in the 67 microbial mat samples from lakes, ponds, streams, seepages, wetlands and wet rock at James Ross Island, including all‐*trans* retinoic acid (ATRA), 13‐*cis* and 9‐*cis* retinoic acid (13‐cis/9‐cis RA), all‐*trans* 4‐hydroxy retinoic acid (4‐OH ATRA), all‐*trans* 4‐keto retinoic acid (4‐keto ATRA), all‐*trans* 5,6‐epoxy retinoic acid (5,6‐epoxy ATRA), retinal, 4‐keto retinal, and 13‐*cis*‐keto retinoic acid (13‐cis keto RA). The concentration of retinoids ranged from undetectable levels (LOD = 1.25 ng/g dm) to concentrations as high as 2.57 μg/g dm of retinal (Figure 2). The most abundant and frequently occurring retinoids were retinal and 4‐keto retinal which were detected in samples from 66 and 52 localities, respectively. On the other hand, 9‐*cis* keto retinoic acid (RA) was not detected (concentrations below LOD), and compounds 13‐cis and 9‐cis RA were detected only at trace concentrations below LOQ (< 2.5 ng/g dm), and therefore not quantified (Table S3).

### 3.2 Distribution of Cyanotoxins Across Different Microbial Mats With Diverse Taxonomic Composition

The taxonomic composition of phototrophs was studied using light microscopy in vertically stratified mats, nonstructured Nostoc‐like mats, and algal filamentous mats sampled across wetlands, streams, ponds, lakes, and wet rocks. These samples were dominated by cyanobacteria, green algae, and diatoms. Vertically stratified mats, the most commonly represented mat type (43 samples), were predominantly dominated by cyanobacteria from the genera Leptolyngbya (14 samples), Oscillatoria (Sehnal 2015), and other cyanobacteria (Codd et al. 1999), whilst a substantial portion of these mats was dominated by diatoms (Kubickova et al. 2019). Besides, the nonstructured Nostoc‐like mats (13 samples) were uniformly dominated by the genus Nostoc (Kubickova et al. 2019), and algal filamentous mats were dominated by green algae (Codd et al. 1999).

**FIGURE 3:**
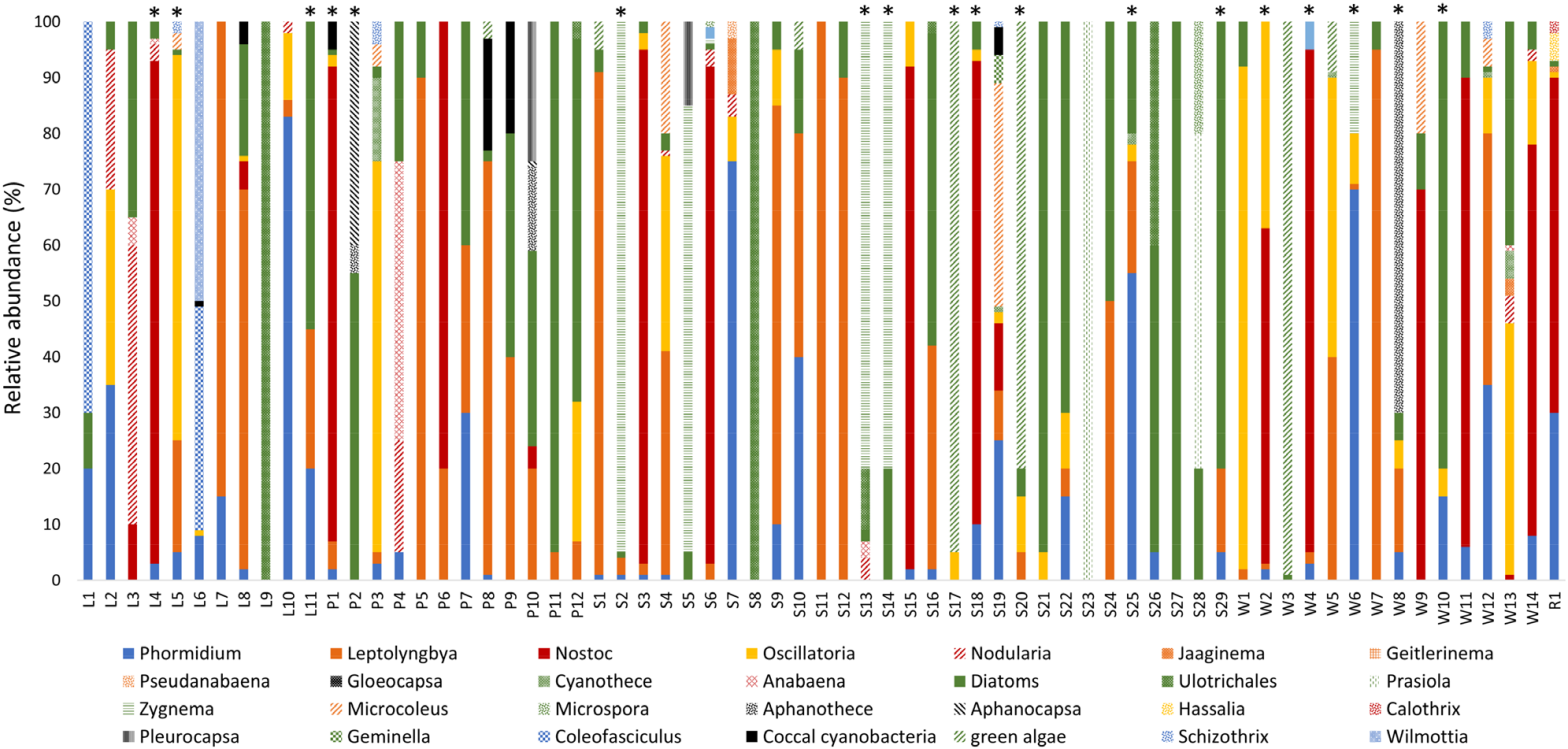
Results of morphological analysis of phototrophic organisms and possible cyanotoxin producers. Relative composition of phototrophic organisms in individual samples. Asterisks indicate a sample with detected cyanotoxin. Detailed concentrations of cyanotoxins are presented in Table 1. L—lake, P—pond, S—stream, W—wetland, R—wetrock. Pictures of dominant taxa from samples with detected cyanotoxins are shown in Figure S3.

Furthermore, Figure 3 shows the abundance of cyanobacteria across individual samples and information about the presence/absence of cyanotoxins. The most common cyanotoxin type, microcystin, was detected in samples with different taxonomic compositions and across all ecosystem types, whilst nodularin was detected in samples strongly dominated by cyanobacteria, specifically from the genus Nostoc. Interestingly, saxitoxin was detected in two samples from streams dominated by green algae, and no cyanobacteria were detected in one of these samples. Due to the absence of genomic data within this study, the nondetection of cyanobacteria in this sample is likely due to the limitations of morphological determination by light microscopy, which can omit low‐abundant species. Deoxy‐cylindrospermopsin was detected only in one sample from the wetland, dominated by cyanobacteria from the genus Leptolyngbya.

## 4 Discussion

Although the number of studies focused on the Antarctic microbial communities, their structure, composition, diversity, and metabolic potential increased over the last decade (de Scally et al. 2016; Koo et al. 2017), ecophysiological interactions and chemical diversity within these communities are still considerably underexplored. Earlier studies reported hepatotoxic microcystins (Wood et al. 2008) and cylindrospermopsin (Kleinteich et al. 2014) in Antarctic microbial communities. The concentrations of microcystins and cylindrospermopsin observed in this study are comparable to those reported by a previous study of Antarctic microbial mats (Kleinteich et al. 2014), where microcystin levels ranged from 10 to 300 ng/g and cylindrospermopsin from 2 to 156 ng/g, but they are relatively lower than the values reported in other studies, where microcystin concentrations in the mats ranged from 500 to 16,000 ng/g dm (Jungblut et al. 2018). However, to our best knowledge, this study provides the first evidence of neurotoxic saxitoxins occurrence in Antarctica.

### 4.1 Cyanotoxins Occurrence, Distribution and Possible Threat to Antarctic Animals

Cyanotoxins were detected in various ecosystems, suggesting their omnipresence across Antarctic ecosystems. Although our results document the widespread distribution of microcystin and point distribution of other cyanotoxins, namely nodularin, cylindrospermopsin, and saxitoxin, across Antarctic ecosystems, the ecological significance of cyanotoxins, not only in the Antarctic, is largely unknown. Earlier studies suggested the possible environmental significance of cyanotoxins in aquatic ecosystems (Metcalf and Codd 2012; Holland and Kinnear 2013). So far, two anticipated ecophysiological roles of cyanotoxins are a competitive advantage or core physiological functions (Holland and Kinnear 2013); however, the physiological roles of cyanotoxins, such as nutrient uptake, maintenance of homeostasis, and protection against oxidative stress, seem to be particularly relevant in Antarctic ecosystems (de Freitas et al. 2024).

From the perspective of possible threats to Antarctic animals, all detected cyanotoxins can have adverse effects on Antarctic vertebrates and invertebrates. The widespread distribution of microcystin across different Antarctic ecosystems and different types of microbial communities is worth a more detailed investigation of the real impact on Antarctic fauna. Interestingly, the first detection of saxitoxin in the Antarctic is a concern for scientists who use the Antarctic water as a source of drinking water since serious neurotoxic effects of saxitoxin have been reported at a concentration of 1 ng/L (Romero‐Alfano et al. 2024). Both samples, where saxitoxin was detected in our study, were collected near Monolith Lake. This location is 10 km distant from the Czech Antarctic station. Therefore, regarding the current threat to members of scientific expeditions to the Czech Antarctic station, there is no data indicating contamination of the water source used for the operation of the station by cyanotoxins.

A key factor contributing to the more widespread distribution of cyanotoxins at hazardous concentrations is climate change, particularly rising temperatures and the prolongation of the vegetative season (Gámez and Manning 2025; Huisman et al. 2018). As a result, cyanotoxin levels in Antarctica may be undergoing significant shifts, especially since 2015, when the samples analysed in this study were collected. However, the role of climate change in altering cyanotoxin dynamics remains largely unexplored in this region. Although long‐term, direct links between climate change and cyanotoxin production in Antarctica remain limited, recent multi‐decadal analyses indicate that climate‐driven changes in temperature, productivity, and freshwater inputs may influence harmful algal bloom dynamics and potentially cyanotoxin occurrence (Brito‐Echeverría et al. 2025). With the results obtained in this study serving as a baseline, this important issue will be studied in greater detail using the samples collected in seasons 2022 and 2025 at James Ross Island, Antarctica.

In addition to cyanotoxins, we detected a variety of different retinoids. Retinoids are derivatives of carotenoids important for various developmental and metabolic processes in animals. An imbalance in retinoid levels can result in severe developmental defects (Pipal et al. 2020). The production of various retinoids by cyanobacteria has already been described (Sehnal et al. 2019; Wu et al. 2012), but only for freshwater planktonic blooms dominated by cyanobacteria. Thus, our study provides the first report of retinoids in benthic mats not only from the Antarctic but from all over the world. Although retinoids are potentially harmful to vertebrates as well as invertebrates (Jonas et al. 2015; Pipal et al. 2020), these cyanobacterial metabolites are unlikely to pose a significant risk to exposed organisms in Antarctic ecosystems, such as birds or numerous invertebrates, at the detected concentrations. Moreover, the individual retinoids significantly differ in their potency to interact with retinoic acid receptors (RARs), where ATRA is the most potent and retinal is the least potent retinoid. The most abundant retinoid in most of the samples is retinal, supporting the prediction for low risk of teratogenic effects caused by retinoids in Antarctic wildlife. Nevertheless, the widespread presence of these compounds emphasises their ecophysiological importance to cyanobacteria. Supported by the results of Sehnal et al. (2022), the role of retinoids can be crucial in oxidative stress mitigation elicited by reactive oxygen species (ROS). Retinoids are end products of ROS‐mediated degradation of carotenoids and apocarotenoids and can protect photoautotrophs in Antarctica against multiple oxidative stresses such as high light stress, UV‐induced stress, or ROS generated during freeze–thaw cycles.

### 4.2 Cyanotoxin Production Informed by Taxonomic Composition

Microscopic analysis of phototrophic members of cyanotoxin‐producing microbial mats brought important information about possible cyanotoxin producers. Whilst microcystin can be produced by a wide range of cyanobacteria from different taxonomic orders (e.g., Nostocales, Oscillatoriales, Synechococcales), the widespread distribution of microcystin across microbial mats dominated by different cyanobacteria is an expected result. In contrast, nodularin production was proved only for species from the genus Nodularia and by symbiotic Nostoc. In our analysis, we detected nodularin in microbial mat dominated by Nostoc (W4), aligning with a previous study (Gehringer et al. 2012), but the determination of the specific producer was out of the scope of this study. The other potent cyanotoxin, deoxy‐cylindrospermopsin, was also detected only in one microbial mat (W6) dominated by species from the genus Leptolyngbya (Synechococcales) with a minor representation of Oscillatoria and Phormidium (Oscillatoriales). Whilst Oscillatoriales species are well‐known cylindrospermopsin producers, no studies have reported Leptolyngbya species as producers of cylindrospermopsin. On the other hand, a member of Synechococcales (Synechococcus) was already identified as a cylindrospermopsin producer (Gin et al. 2021). Nevertheless, regarding the taxonomic distance of Synechococcus and Leptolyngbya, and the low concentration (below LOQ) of deoxy‐cylindrospermopsin detected in the sample, the production of this cyanotoxin by Oscillatoriales species is a more likely scenario. Although the above‐discussed cyanotoxins are connected to a possible cyanobacterial producer, the producer of saxitoxin detected in this study is unknown. The samples where saxitoxin was detected are dominated by green algae (Chlorophyta). Saxitoxin producers are found primarily within the order Nostocales, including genera such as *Anabaena, Dolichospermum*, *Aphanizomenon, Cuspidothrix*, *Raphidiopsis, Cylindrospermopsis*, *Cylindrospermum* and *Scytonema*. Additionally, representatives from the order Oscillatoriales, such as *Geitlerinema*, *Limnothrix*, *Microseira*/*Lyngbya*, *Planktothrix*, and *Phormidium*, have also been reported as potential saxitoxin producers (Christensen and Khan 2020; Bernard et al. 2016). However, the only cyanobacteria detected in one of those saxitoxin‐positive samples, *Oscillatoria* sp., has not been reported as a saxitoxin producer. Therefore, the identification and potential isolation of the actual saxitoxin‐producing strain from the Antarctic mats remains an open task. The absence of cyanobacteria in one saxitoxin‐positive sample is likely due to limitations of morphological identification. Since the saxitoxin could be hazardous to researchers who work on James Ross Island, the specific producer of saxitoxin in these Antarctic samples should be studied in future with the application of molecular biology techniques to detect saxitoxin biosynthetic genes and analysis of their taxonomic origin. Alarmingly, the production of saxitoxin can be an even bigger issue as a consequence of profound climate change in Antarctica and poses a threat to Antarctic wildlife, including birds and penguins.

### 4.3 Limitations of the Study

The first weakness is the absence of sequencing analysis of 16S and 18S rRNA genes for detailed taxonomic analysis, together with PCR detection and sequencing of conserved genes from the saxitoxin biosynthetic gene cluster. This information would allow the specific identification of the taxonomic producer of saxitoxin. The second weakness of the study is the lack of collection of water samples to analyse the saxitoxin presence in free water, to bring more relevant information towards the real risk of saxitoxin for scientists and other Antarctic organisms exposed to the free water containing saxitoxin. These analyses will be involved in a subsequent targeted study.

## 5 Conclusions

The findings presented herein contribute not only to the growing body of knowledge on cyanotoxins but also to the broader scientific discourse surrounding the biological hazard and risk in the most remote ecosystems of our planet. This study brings new information about (i) the widespread distribution of cyanotoxins across all aquatic ecosystems in Antarctica, (ii) the first data about concentrations of retinoids, proven EDCs, in Antarctica, and (iii) the first report about the detection of the highly potent neurotoxin saxitoxin in Antarctica. The emergence of saxitoxin in Antarctica raises critical questions about the factors influencing its presence and the potential impacts on local and broader ecological consequences within this unique ecosystem.

## Author Contributions

L.S. collected samples, processed the samples, including chemical extraction, carried out taxonomic classification, designed the study, and wrote and edited the manuscript. M.S. participated in the sample processing and manuscript preparation. L.B. carried out the L.S.‐M.S. analysis and participated in manuscript preparation. P.B. participated in chemical analysis and manuscript preparation. O.M. participated in data analysis and manuscript preparation. K.H. participated in study design, manuscript preparation, and editing.

## Funding

This work was supported by the Ministry of Education (CZ.02.1.01/0.0/0.0/17_043/0009632, LM2023069), Grant Agency of Masaryk Univerzity (MUNI/JS/1964/2025), Horizon 2020 Framework Programme (857560), and Institute of Botany of the Czech Academy of Sciences (RVO 67985939).

## Conflicts of Interest

The authors declare no conflicts of interest.

## Supporting information


**Data S1:** Supporting Information 1.

**Data S2:** Supporting Information 2.

## Data Availability

The data that supports the findings of this study are available in the Supporting Informations of this article.
