# Supplementary material for: Occurrence and Diversity of Cyanotoxins and Retinoid Compounds in Antarctic Microbial Mats: Evidence From James Ross Island
Source: Environ Microbiol Rep. 2026 Mar 31;18(2):e70321. doi: 10.1111/1758-2229.70321 (PMC13086568; doi:10.1111/1758-2229.70321)
Supplement: Supplementary file 1 — Data S1: Supporting Information 1. [file EMI4-18-e70321-s001.docx]

**Supplementary Material**

# Supplementary Material S1 - List of chemicals and reagents

Standard all-trans-Retinoic acid (ATRA, ≥98%), 13-cis-Retinoic acid (13-cis RA, ≥98%) were obtained from Sigma-Aldrich (Merck, Rahway, NJ), 9-cis-Retinoic acid (9-cis RA, ≥98%) from Santa Cruz Biotechnology (Dallas, TX) and all trans-Retinal (retinal), rac all- trans 4-Hydroxy Retinoic acid (4OH-ATRA), all-trans 4-Keto Retinoic acid ( 4keto-ATRA), 4-Keto Retinal (4keto-retinal), all-trans 5,6-Epoxy Retinoic Acid (5,6epoxy-ATRA), 4-Keto 13-cis-Retinoic Acid (4keto-13cis-RA), 4-Keto 9-cis-Retinoic Acid (4keto-9cis-RA) from Toronto Research Chemicals (North York, Canada) which do not provide % purity. Labelled standards of ATRA-d5, retinal-d5, 4keto-ATRA -d3 and 4keto-13cis-RA -d3 were purchased from Toronto Research Chemicals.

Standards of anatoxin (ATX, ≥98%) and saxitoxin dihydrochloride solution (STX, 20mg/g) were obtained from Cayman Chemical (Ann Arbor, MI) and Sigma-Aldrich (Merck) and cylindrospermopsin (CYN, 95%), microcystin WR (MC-WR, 95%) and the mixture of microcystin LR (MC-LR), microcystin RR (MC-RR), microcystin LA (MC-LA), microcystin LF (MC-LF), microcystin LW (MC-LW), microcystin YR (MC-YR), microcystin LY (MC-LY) and Nodularin (NOD) at 95% purity were obtained from Novakits (Nantes, France). Labeled standards of MC-RR-15N13 (98%), MC-LR-15N10 (98%), L-phenylalanine (Phe-D5, 98%) were purchased from Cambridge Isotope Laboratories, Inc. (Tewksbury, MA) and [15N5] cylindrospermopsin (CYN-15N5, ~100) was purchased from Eurofins Abraxis (Warminster, PA).

**Supplementary Material S2 – Analytical methods for retinoids**

Analysis was performed with a Waters LC chromatograph (Waters, Manchester, U.K.) and the chromatographic separation was achieved using a column Acquity BEH C18 of 100 x 2.1 mm ID and 1.7 μm particle size. A gradient elution method was set with a phase A (0.1% formic acid in water) and phase B (0.1% formic acid in acetonitrile). MS grade acetonitrile and formic acid (FA, 99%) were purchased from BIOSOLVE BV (Valkenswaard, Netherlands).

The gradient elution started with 20% B, increased to 70% B over 1 min, and to 100%B from 1 to 5 min, held (100% B) from 5 to 7 min, then decreased to 20% B in 7 min and held for 2 min to equilibrate the system before the next injection. The flow rate was set at 0.3 mL/min and the injection volume at 7 μL. The column and sample temperatures were set at 40°C and 10°C, respectively.

Detection was performed on a Xevo TQ-S quadrupole mass spectrometer (Waters) and analytes in ESI+ mode were detected using tandem mass spectrometry. The collision energy of transitions (ATRA, 9cis-RA, 13cis-RA, retinal, 4keto-atRA, 13-cis-oxoRA, 9-cis-oxoRA, 5,6epoxy-atRA and 4keto-retinal; 9-cis RA and 13-cis RA were reported as a sum) were optimized for each analyte (Table S4). The capillary voltage was set at 0.8 kV and the cone, desolvation and collision gas flows were set at 150 (L/h), 600 (L/h) and 0.15 (mL/min), respectively. The source and desolvation temperature were set at 150°C and 500°C, respectively. Data were processed by MassLynxTM software (Waters). The quantification of analytes was based on the most intensive MRM transition, considering relative signals between each analyte and internal standard (4keto atRA-D3; retinal-D5; atRA-D5; 13cis-oxoRA-D3). The limit of detection (LOD, signal to noise ratio S/N>3) and quantification (LOQ, S/N>10) were 0.5 and 1 ng/mL, respectively for each compound.

**Supplementary Material S3 – Analytical methods for cyanotoxins**

Analyses of cyanotoxins were performed with Waters Acquity LC chromatography (Waters) and the chromatographic separation was achieved using a column an Acquity UPLC BEH C18 (1.7 μm) (Waters) 100 x 2.1 mm equipped with a guard precolumn kept at 35°C. Detection was performed on a Xevo TQ-S quadrupole mass spectrometer (Waters). Analytes after ESI ionization were detected in positive ion mode using tandem mass spectrometry with multiple reaction monitoring (MRM).

The mobile phase consisted of 0.1% formic acid in water (A) and acetonitrile acidified with 0.1% formic acid (B). MS grade acetonitrile and formic acid (FA, 99%) were purchased from BIOSOLVE BV. The binary pump gradient was: 2%B 0.3mL/min at 0-1min, 2%-70%B 0.45ml/min at 1-9min, 90%B 0.45ml/min at 9-10min followed by 3 min column equilibration to the initial conditions 0.3mL/min (2%B). The injection was 7 μL of the individual sample from the thermostatted autosampler (10°C). The ionization parameters were as follows: capillary voltage, 3kV; the source temperature and the desolvation temperature, 150 and 550 °C, respectively; the cone gas flow, 150 (L/h); the desolvation gas flow, 800 (L/h); and the collision gas flow, 0.15 mL/min. The collision energy was optimized for each analyte (MC-LR, RR, YR, LY, LW, WR, LA, LF, NOD, CYN, ATX, STX (Table S5). Data were processed by MassLynxTM software (Waters). The quantification of analytes was based on the most intensive MRM transition, considering relative signals between each analyte and internal standard (MC-RR-15N13, MC-LR-15N10, CYN-15N5, Phe-D5). The limit of detection for biomass extract was (S/N>3) 0.5 ng/mL, and the limit of quantification (S/N>10) 1 ng/mL for each analyte.

**Table S1.** List of samples, collection conditions, and GPS coordinates (Excel file: Supplementary materials - Tables).

**Table S2.** The concentration of analyzed cyanotoxins in Antarctic microbial mats. Empty box indicates that the compound was not detected in a concentration above LOD. Asterix indicates that the compound was detected in concentration between LOD and LOQ (Excel file: Supplementary materials - Tables).

**Table S3.** Concentration of analyzed retinoids in Antarctic microbial mats. Empty box indicates that the compound was not detected in a concentration above LOD. Asterix indicates that the compound was detected in concentration between LOD and LOQ (Excel file: Supplementary materials - Tables).

**Table S4.** MS parameters for analyzed retinoic compound; MRM (multiple reaction monitoring); quantification ions marked (bold)

| Compound | MRM transition | Cone voltage (V) | Collision energy (V) |
| --- | --- | --- | --- |
| 4-OH atRA | **299.2 → 95.2** | 30 | 23 |
|  | 299.2 → 157.2 | 30 | 25 |
| 4-keto atRA | **315.2 → 137.1** | 30 | 25 |
|  | 315.2 → 241.2 | 30 | 15 |
| 4-keto retinal | **299.2 → 147.2** | 30 | 24 |
|  | 299.2 → 189.2 | 30 | 16 |
| 5.6-epoxy RA | **317.2 → 107.2** | 30 | 21 |
|  | 317.2 → 153.2 | 30 | 14 |
| 9-cis RA/13-cis RA | **301.2 → 159.1** | 30 | 23 |
|  | 301.2 → 205.1 | 30 | 13 |
| atRA | **301.2 → 159.1** | 30 | 23 |
|  | 301.2 → 205.1 | 30 | 13 |
| retinal | **285.3 → 161.2** | 30 | 9 |
|  | 285.3 → 175.2 | 30 | 13 |
| 13-cis-oxoRA | **315.3 → 137.1** | 30 | 25 |
|  | 315.3 → 241.2 | 30 | 15 |
| 9-cis-oxoRA | **315.3 → 137.1**  315.3 → 241.2 | 30  30 | 25  15 |
|  |  |  |  |
|  |  |  |  |
| atRA-d5 | **306.2 → 162.2** | 30 | 20 |
|  | 306.2 → 206.2 | 30 | 15 |
| retinal-d5 | **290.3 → 161.2** | 30 | 8 |
|  | 290.3 → 180.3 | 30 | 15 |
|  | 290.3 → 198.2 | 30 | 7 |
| 4keto atRA-d3 | **318.2 → 137.2** | 30 | 24 |
|  | 318.2 → 162.2 | 30 | 14 |
|  | 318.2 →244.2 | 30 | 14 |
| 13-cis-oxoRA-d3 | **321.2 →143.1** | 30 | 25 |
|  | 321.2 →247.2 | 30 | 15 |
|  |  |  |  |

**Table S5.** MS parameters for analyzed cyanotoxins; MRM (multiple reaction monitoring); quantification ions marked (bold)

| Compound | MRM transition | Cone voltage (V) | Collision energy (V) |
| --- | --- | --- | --- |
| ATX | **166.1 → 43.0** | 35 | 20 |
|  | 166.1 → 91.0 | 35 | 10 |
|  | 166.1 → 131.1 | 35 | 14 |
| DATX | **168.0 → 133.0** | 35 | 17 |
|  | 168.0 → 150.1 | 35 | 17 |
| HATX | **180.0 → 163.1** | 35 | 15 |
|  | 180.0 → 145.1 | 35 | 18 |
| DHATX | **182.0 → 147.0** | 35 | 17 |
|  | 182.0 → 164.1 | 35 | 17 |
| STX | **300.0 → 204.0** | 45 | 25 |
|  | 300.0 → 138.0 | 45 | 25 |
|  | 300.0 → 179.0 | 45 | 25 |
| 7-deoxyCYN | **400.0 → 194.1** | 55 | 35 |
|  | 400.0 → 320.0 | 55 | 40 |
| CYN | **416.2 → 194.2** | 60 | 38 |
|  | 416.2 → 176.0 | 50 | 20 |
|  | 416.2 → 336.2 | 61 | 22 |
| MC-RR  MC-LA  MC-LF  MC-LR  MC-LY  MC-LW  MC-YR  MC-WR  NOD | **520.2 → 135.1**  520.2 → 70.0  520.2 → 127.0  **911.5 → 135.1**  **986.6 → 135.1**  986.6 → 249.2  **995.5 → 135.1**  995.5 → 213.0  995.5 → 107.1  **1002.7 → 135.1**  1002.7 → 107.1  **1025.7 → 135.1**  1025.7 → 127.1  **1045.7 → 135.1**  1045.7 → 127.1  1045.7 → 213.0  **1068.5 → 135.1**  **825.4 → 135.1**  825.4 → 227.2 | 60  60  60  60  60  60  60  65  60  60  60  60  60  60  62  55  60  80  80 | 28  50  30  70  60  50  70  60  80  72  76  70  80  70  85  60  75  60  50 |
|  |  |  |  |
| MC-RR-15N13  MC-LR-15N10 | **526.2 → 135.1**  **1005.6 → 135.0** | 60  65 | 40  70 |
| CYN-15N5 | **421.1 → 341.2** | 60 | 20 |
|  |  |  |  |
| Phe-D5 | **171.1 → 125.2** | 35 | 17 |
|  |  |  |  |

**
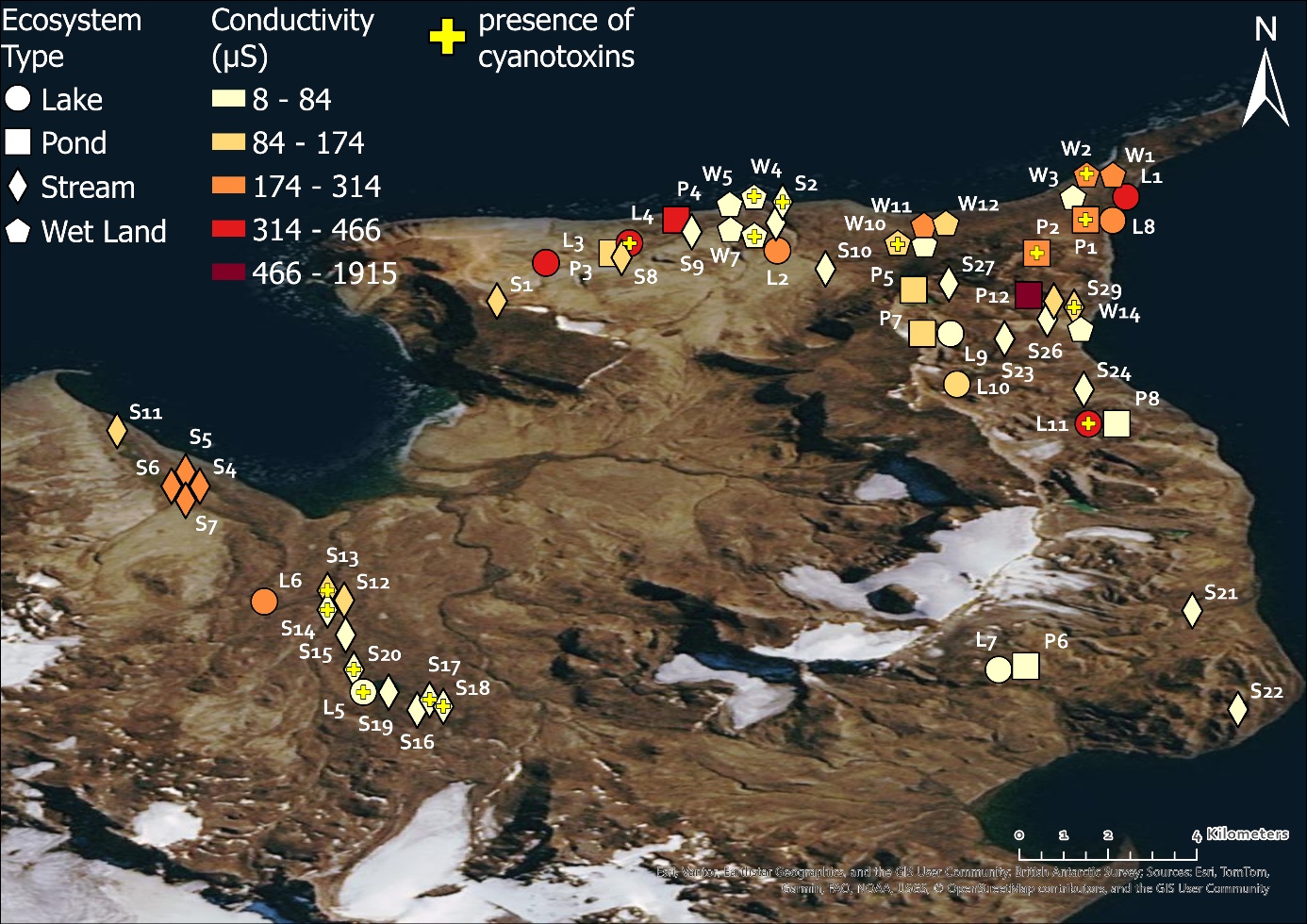
**

**Figure S1.** Map of James Ross Island and types of sampling sites with indicated presence of cyanotoxin and localities coloured based on conductivity gradient. Selected site markers have been offset from their exact positions to avoid overlap on the map. Accurate coordinates for each sampling site are provided in the accompanying sample table (Table S1).

**
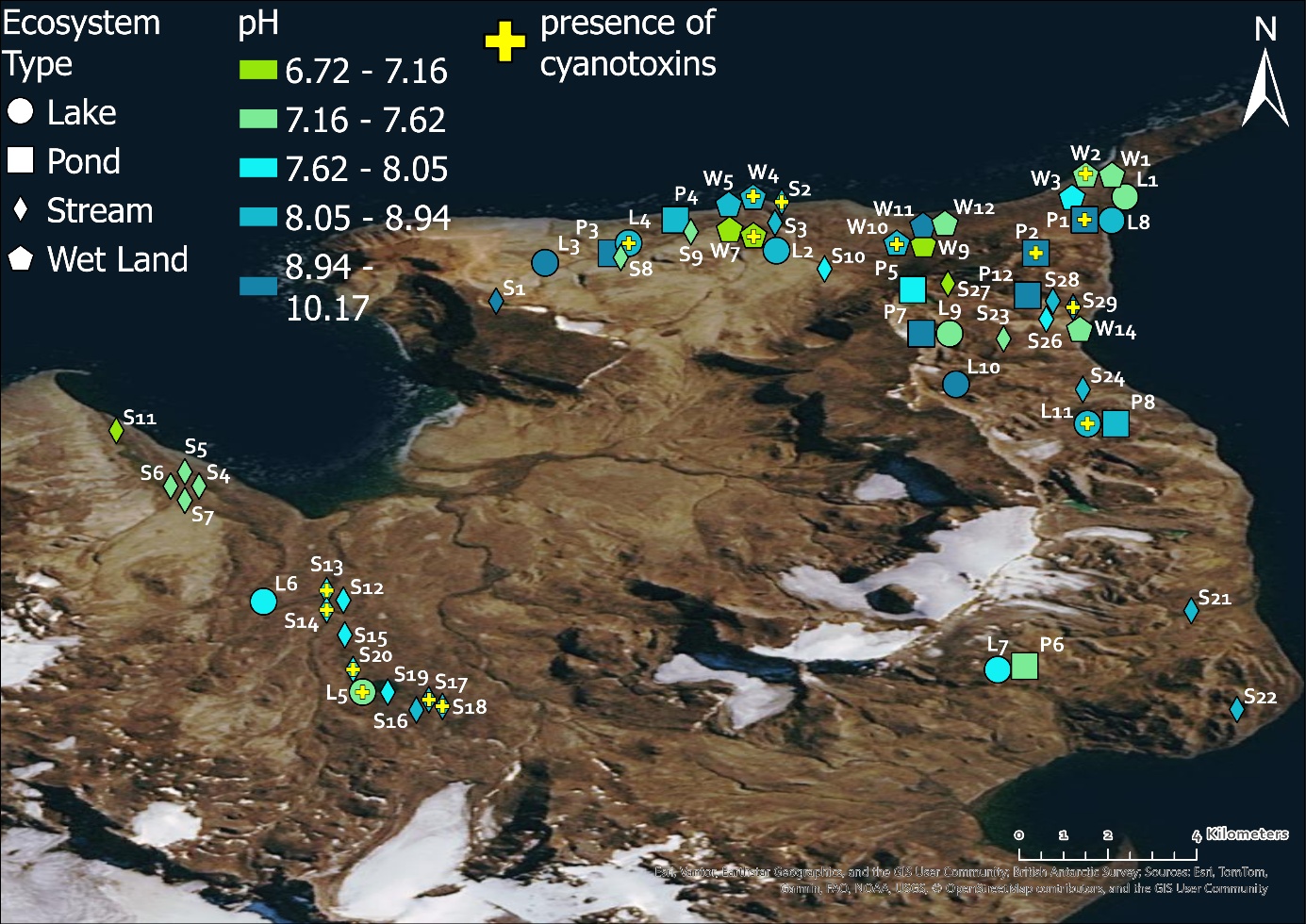
**

**Figure S2.** Map of James Ross Island and types of sampling sites with indicated presence of cyanotoxin and localities coloured based on pH gradient. Selected site markers have been offset from their exact positions to avoid overlap on the map. Accurate coordinates for each sampling site are provided in the accompanying sample table (Table S1).

**
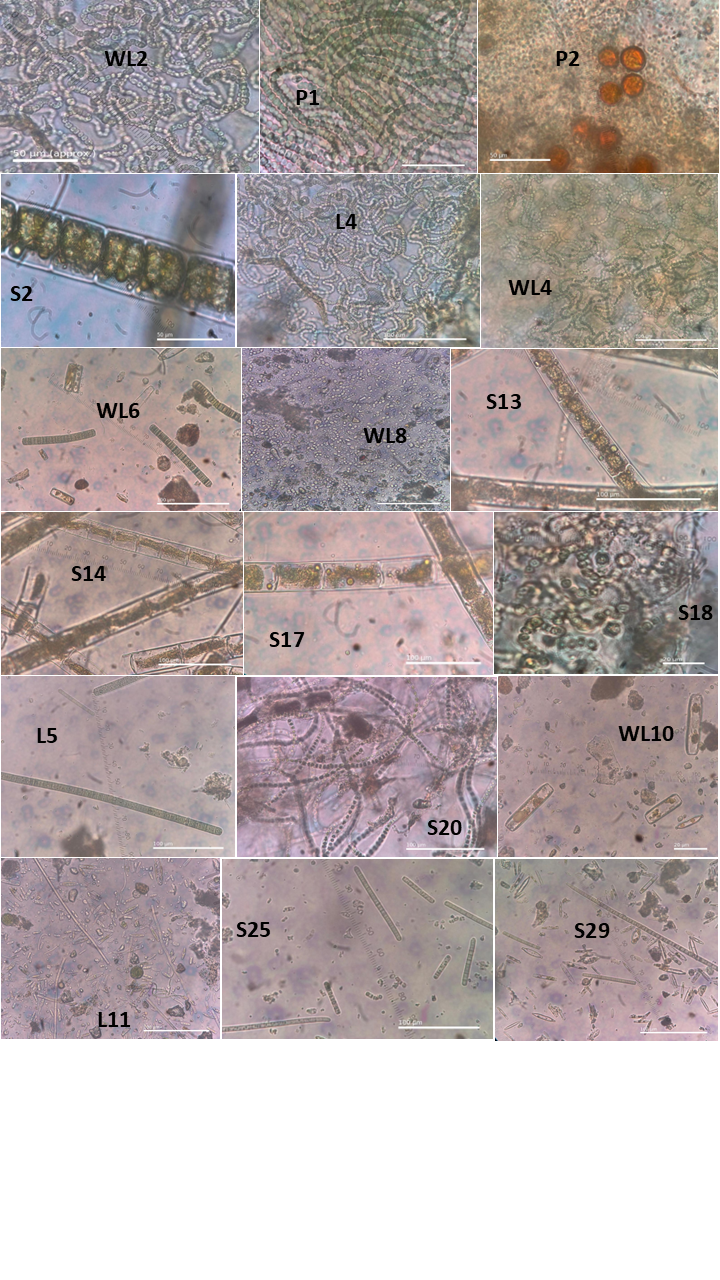
**

**Figure S3.** Pictures of dominant photoautotrophs from samples with detected cyanotoxins. Dominant species: W2 – Nostoc sp., P1 - Nostoc sp., P2 – Aphanocapsa (green coccoid cells, not orange cells), S2 – Zygnema sp., L4 – Nostoc sp., W4 – Nostoc sp., W6 – Phormidium sp., W8 – Aphanothece, S13 – Zygnema sp., S14 – Zygnema sp., S17 – Zygnema sp., S18 – Nostoc sp., L5 – Phormidium sp., S20 – Ulotrichales, W10 – Diatoms, L11 – Diatoms, S25 – Phormidium, S29 – Diatoms. L – lake, P – pond, S – stream, W – wetland.
